# Automated detection of diabetic retinopathy lesions in ultra-widefield fundus images using an attention-augmented YOLOv8 framework

**DOI:** 10.3389/fcell.2025.1608580

**Published:** 2025-07-24

**Authors:** Lei-Si Hu, Jie Wang, Heng-Ming Zhang, Hai-Yu Huang

**Affiliations:** ^1^First Clinical College, Chongqing Medical University, Chongqing, China; ^2^ Eye School of Chengdu University of Traditional Chinese Medicine, Chengdu, Sichuan, China; ^3^School of Computer and Artificial Intelligence, Southwest Jiaotong University, Chengdu, Sichuan, China

**Keywords:** diabetic retinopathy, YOLOv8, ultra-widefield fundus images, automatic detection, attention mechanisms

## Abstract

**Objective:**

To enhance the automatic detection precision of diabetic retinopathy (DR) lesions, this study introduces an improved YOLOv8 model specifically designed for the precise identification of DR lesions.

**Method:**

This study integrated two attention mechanisms, convolutional exponential moving average (convEMA) and convolutional simple attention module (convSimAM), into the backbone of the YOLOv8 model. A dataset consisting of 3,388 ultra-widefield (UWF) fundus images obtained from patients with DR, each with a resolution of 2,600 × 2048 pixels, was utilized for both training and testing purposes. The performances of the three models—YOLOv8, YOLOv8+ convEMA, and YOLOv8+ convSimAM—were systematically compared.

**Results:**

A comparative analysis of the three models revealed that the original YOLOv8 model suffers from missed detection issues, achieving a precision of 0.815 for hemorrhage spot detection. YOLOv8+ convEMA improved hemorrhage detection precision to 0.906, while YOLOv8+ convSimAM achieved the highest value of 0.910, demonstrating the enhanced sensitivity of spatial attention. The proposed model also maintained comparable precision in detecting hard exudates while improving recall to 0.804. It demonstrated the best performance in detecting cotton wool spots and the epiretinal membrane. Overall, the proposed method provides a fine-tuned model specialized in subtle lesion detection, providing an improved solution for DR lesion assessment.

**Conclusion:**

In this study, we proposed two attention-augmented YOLOv8 models—YOLOv8+ convEMA and YOLOv8+ convSimAM—for the automated detection of DR lesions in UWF fundus images. Both models outperformed the baseline YOLOv8 in terms of detection precision, average precision, and recall. Among them, YOLOv8+ convSimAM achieved the most balanced and accurate results across multiple lesion types, demonstrating an enhanced capability to detect small, low-contrast, and structurally complex features. These findings support the effectiveness of lightweight attention mechanisms in optimizing deep learning models for high-precision DR lesion detection.

## 1 Introduction

Diabetes mellitus (DM) is a pervasive chronic disease that imposes a substantial challenge on global public health systems. Diabetic retinopathy (DR) is recognized as a leading cause of vision impairment among working-age adults (Flaxel et al., 2020). The number of individuals diagnosed with DM worldwide is estimated to reach 700 million by the year 2045 (Saeedi et al., 2019). Elevated blood glucose levels in individuals with DM impose retinal vascular damage, which can lead to vision loss and the onset of DR (Randive et al., 2019). Over 60% of patients with type-2 DM will experience some degree of DR within 20 years (Keenan et al., 2007). DR is often asymptomatic in its initial stages. However, damage to the optic nerve and microvascular alterations, such as microaneurysms and hemorrhages, may already be present (Ruan et al., 2022). By the time patients seek treatment for vision impairment, fundoscopic examinations often reveal advanced retinal damage. Consequently, the early screening, diagnosis, and treatment of DR are of paramount importance (Oh et al., 2021; Wu et al., 2017).

With the rapid advancement of artificial intelligence (AI)-based diagnostic technologies, AI is transforming the landscape of ophthalmic healthcare. In recent years, China has made significant strides in AI-driven ophthalmology, with expert groups formulating multiple clinical guidelines and developing a variety of intelligent diagnostic models across ophthalmic subspecialties. These models are gradually being integrated into routine clinical practice (Gong et al., 2024). In the field of intelligent DR diagnosis, numerous research groups, both in China and internationally, have developed a variety of AI-based diagnostic models, all of which have demonstrated outstanding performance. In 2018, the U.S. Food and Drug Administration (FDA) approved IDx-DR as the first autonomous AI system for the diagnosis of DR (Abràmoff et al., 2018). Khan et al. (2025) conducted a systematic review and meta-analysis to evaluate the diagnostic accuracy of IDx-DR. A total of thirteen studies involving 13,233 participants met the inclusion criteria. The results showed a pooled sensitivity of 0.90 and a pooled specificity of 0.91 for IDx-DR. The area under the summary receiver operating characteristic (ROC) curve was 0.95, indicating a high level of diagnostic accuracy. Gulshan et al. (2016) developed a deep learning algorithm (DLA) that can automatically detect vision-threatening referable DR. In the internal validation dataset, the model achieved an area under the curve (AUC) of 0.989, with a sensitivity of 97.0% and a specificity of 91.4%. Arsalan et al. (2019) proposed a diagnostic model based on VesselNet and validated its performance in diagnosing DR using three public datasets: DRIVE, CHASE-DB1, and STARE. For the DRIVE dataset, the model achieved a sensitivity of 0.80, a specificity of 0.98, an AUC of 0.98, and an accuracy of 0.96. For the CHASE-DB1 dataset, the corresponding values were 0.82, 0.98, 0.98, and 0.97, respectively; for the STARE dataset, they were 0.85, 0.99, 0.99, and 0.97, respectively. These results demonstrate the model’s exceptional detection performance across multiple datasets. Dai et al. (2021) established a DeepDR system utilizing the residual network (ResNet) architecture. The model was trained on a substantial dataset comprising 466,247 fundus images from patients diagnosed with diabetes mellitus, sourced from three distinct datasets, to facilitate real-time assessments of image quality, lesion detection, and disease grading. The system was validated using 209,322 images, and the results revealed that the AUC values for both lesion detection and grading exceeded 0.9, indicating a strong diagnostic capability. Deng et al. (2024) employed residual U-Net in conjunction with transfer learning techniques to conduct a quantitative analysis of retinal vascular morphology using ultra-widefield (UWF) fundus images. The model demonstrated an accuracy of 99% and a Dice coefficient of 0.76.

However, there are relatively few models that are specifically designed for disease diagnosis using UWF fundus images. Most existing models do not specialize in recognizing features from subtle lesions, such as hemorrhages, hard exudates, and cotton wool spots. Instead, they rely on more generalized features for disease classification, which affects the accuracy and explainability of the model. To overcome this issue, we propose a subtle lesion detection approach for DR in UWF fundus images utilizing the YOLOv8 model.

The YOLO (you only look once) algorithm was first introduced in 2016 at the IEEE Conference on Computer Vision and Pattern Recognition. YOLOv1 (Redmon et al., 2016) adopted a single-stage object detection framework. YOLOv2 (Redmon and Farhadi, 2017) introduced several enhancements, including batch normalization, high-resolution classification training, and anchor boxes for bounding box prediction. YOLOv3 (Redmon, 2018) incorporated multi-scale detection and a ResNet architecture, further improving detection accuracy. YOLOv4 (Bochkovskiy et al., 2020) implemented multiple optimization techniques—such as mosaic data augmentation, complete intersection over union (CIoU) loss, and stochastic weight averaging (SWA)—achieving a better balance between real-time performance and detection precision, thereby establishing itself as one of the leading object detectors. YOLOv5 (Glenn Jocher et al., 2022) introduced automated data learning, enabling the model to dynamically adapt its augmentation strategies based on the input data, thereby enhancing generalization. YOLOv7 (Wang et al., 2023) incorporated the extended efficient layer aggregation network, surpassing YOLOv5 in terms of accuracy, speed, and efficiency. YOLOv8 (Jocher et al., 2023) is a refined version of the YOLO architecture with a more lightweight multi-scale object detection structure, including an improved neck layer and an automatic anchor box adjustment strategy.

Due to the approximately 200° retinal coverage of UWF fundus images, the types of lesions observed are diverse, edge distortion is pronounced, small lesions are numerous, and background interference is significant. Traditional models often demonstrate unstable localization and low recall when applied to these images. To address the class imbalance between lesion and non-lesion regions—particularly in the context of small targets—many object-detection models have adopted enhanced loss functions, such as focal loss (Lin et al., 2020), which has demonstrated exceptional performance in dense detection tasks. Building upon this challenge, the present study integrates two attention mechanisms, convolutional exponential moving average (convEMA) and convolutional simple attention module (convSimAM), into the YOLOv8 backbone to tackle critical issues in lesion detection from ultra-widefield scanning laser ophthalmoscopy (UWF-SLO) images. These issues include the frequent omission of small lesions, indistinct structural boundaries, and disorganized spatial responses. The two attention mechanisms enhance the model’s ability to recognize DR lesions in UWF fundus images from both channel and spatial dimensions. While keeping the model lightweight, these mechanisms significantly improve detection performance and interpretability, thereby providing a more efficient technical foundation for the intelligent screening of DR. Bibliometric evidence indicates that current AI research in DR is increasingly focused on object detection, attention mechanisms, and models suitable for real-time deployment (Wang et al., 2022). This trend underscores the growing demand for lightweight, interpretable, and high-resolution lesion-detection frameworks, particularly in the context of UWF fundus imaging.

## 2 Methods

### 2.1 Dataset

This study retrospectively collected 3,388 fundus images from patients diagnosed with DR who underwent UWF fundus imaging examinations. All images were acquired by experienced ophthalmic technicians and had a resolution of 2,600 mm × 2,048 pixels. The study was thoroughly reviewed and approved by the Institutional Medical Ethics Committee of our hospital (approval no. DQAIER202203001). Since this was a retrospective study utilizing anonymized UWF fundus images with all patient-identifiable information removed, informed consent from participants was not required.

### 2.2 Image processing

**FIGURE 1:**
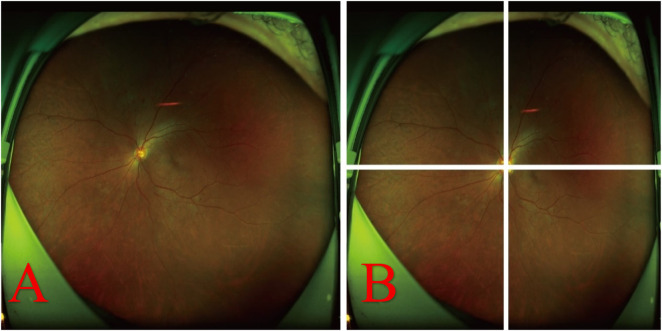
Original fundus image and its optic disc-centered cropping into four sub-images. **(A)** Original image. **(B)** Image centered on the optic disc and cropped into four distinct sub-images.

Due to the frequent presence of non-informative regions such as eyelids and eyelashes in UWF fundus images, these areas increase the computational load during training and provide no value for feature extraction, thereby hindering the detection of DR lesions. Furthermore, when the entire high-resolution UWF fundus image is input into the model, the wide range of pixel values complicates the algorithm’s ability to concentrate on localized lesions. Consequently, during the initial stage of model training, each image was cropped and segmented into four sub-images centered on the optic disc (as illustrated in Figure 1), facilitating more focused and efficient lesion detection.

### 2.3 Lesion annotation

**FIGURE 2:**
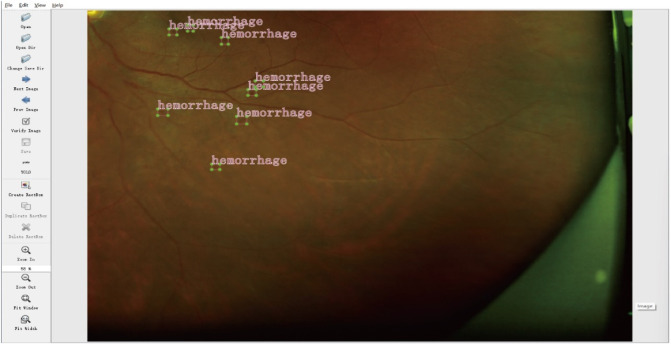
Example of lesion annotation.

The collected UWF fundus images were annotated by two experienced ophthalmologists who independently identified and labeled the DR-related lesions. In instances of disagreement, a third senior ophthalmologist (associate chief physician or higher) reviewed the images and provided guidance to resolve the discrepancies. Manual annotation was conducted using LabelImg software, focusing on key DR lesions, including hemorrhages, hard exudates, cotton wool spots, and epiretinal membranes. Each annotation encompassed spatial coordinates and lesion type labels, as illustrated in Figure 2.

## 3 Model construction and training

### 3.1 YOLOv8 model

The YOLOv8 model consists of three primary components: the backbone, the neck, and the head. The backbone is responsible for feature extraction and is built upon a deep learning architecture optimized for handling fine-grained features and complex backgrounds. It shares similarities with several well-known architectures, including cross stage partial networks (Ge et al., 2021), residual transformation blocks (He et al., 2016), the dense convolutional network (Huang et al., 2017), and the visual geometry group network (Simonyan and Zisserman, 2014). This architecture enables YOLOv8 to effectively capture rich visual representations while maintaining computational efficiency. The neck employs a hybrid structure that integrates the path aggregation network (PAN) and the feature pyramid network (FPN) (Liu et al., 2018; Lin et al., 2017) to fuse multi-scale features, thereby enhancing the model’s capability to detect objects of varying sizes. Additionally, YOLOv8 substitutes the traditional C3 modules with a more efficient C2f module, which improves gradient propagation and minimizes model redundancy. This modification enables the network to more effectively manage small lesions and densely packed features, which are prevalent in UWF fundus images. The detection head in YOLOv8 is responsible for mapping the extracted feature representations to the final outputs, which include bounding box coordinates, category confidence scores, and object classification results. Unlike previous versions that utilized an anchor-based mechanism, YOLOv8 employs an anchor-free strategy. This approach enhances flexibility, reduces computational overhead, and facilitates model training and optimization. This design allows the model to better accommodate objects of varying shapes and sizes.

The introduction of the anchor-free mechanism eliminates the need for manually predefined anchor boxes, thereby simplifying the model design. However, conventional anchor-based approaches, anchor boxes that can encompass a wide range of spatial positions and scales, enhance the likelihood of detecting small targets. In contrast, the anchor-free paradigm may be less effective in this aspect, potentially resulting in suboptimal performance in small object detection, which is an important consideration in medical imaging scenarios where precise lesion localization is critical.

### 3.2 Integration of attention mechanisms

UWF fundus images utilize scanning laser ophthalmoscopy technology in conjunction with elliptical mirror optics. By employing the principle of elliptical dual foci, this technology facilitates rapid retinal imaging over a 200° field of view in just 0.25 s while preserving high resolution (Carles et al., 2017; Fischer et al., 2019). Given the extensive imaging range of UWF fundus images, the lesions in patients with DR are often relatively small. To improve the extraction of lesion-related features, we incorporated two attention mechanisms into the YOLOv8 architecture.

The first module is convEMA (Sheng and Chen, 2023), a convolutional channel attention mechanism that incorporates an exponential moving average (EMA) strategy. By utilizing a learnable EMA approach, convEMA enhances the consistency of channel-wise features and improves the model’s ability to reliably perceive small targets, thereby strengthening its focus and robustness in detecting subtle features such as hemorrhages and microaneurysms. Channel attention modules, such as CBAM (Woo et al., 2018), have been widely adopted to enhance feature representation in deep learning models. In this study, we propose a simplified alternative—convEMA—which stabilizes the learning of DR lesion features with reduced computational overhead.

**FIGURE 3:**
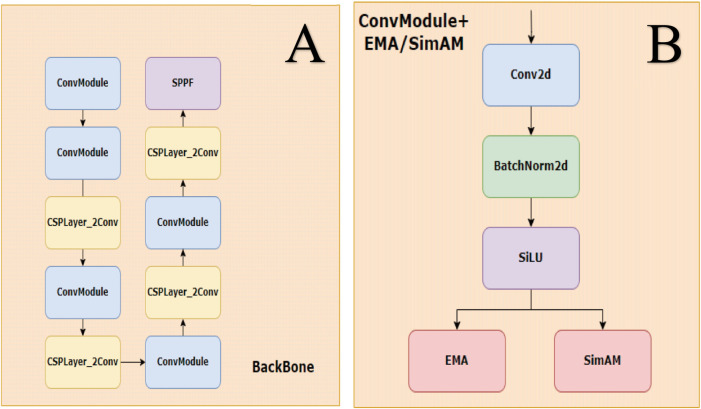
Backbone and ConvModule structure. **(A)** The backbone is composed of stacked ConvModule and CSPLayer_2Conv blocks, ending with an SPPF module for multi-scale feature extraction. **(B)** ConvModule includes Conv2d, BatchNorm2d, and SiLU activation, with optional attention modules (EMA or SimAM) applied after activation.

The second module is convSimAM (Yang et al., 2021), a parameter-free spatial attention approach inspired by the neuronal minimum energy inhibition principle. It calculates the “neural activation energy” at each spatial location within the input feature map, facilitating enhanced spatial focus on lesion regions with ambiguous boundaries without increasing the training burden. Unlike conventional local attention modules, non-local neural networks (Wang et al., 2018) explicitly model long-range spatial dependencies. Similarly, convSimAM improves the model’s capacity to capture spatial cues in blurry or low-contrast DR lesions. Its lightweight architecture and parameter-free design render it particularly well-suited for real-time detection tasks. Notably, it is especially effective for spatial modeling of weak-boundary targets in complex background images, such as epiretinal membranes and cotton wool spots. The structural design of the modified architecture is illustrated in Figure 3.

By combining channel-wise and spatial-wise attention, the modified YOLOv8 framework significantly boosts lesion detection performance without compromising computational efficiency. This integration is particularly beneficial for medical imaging tasks characterized by high-resolution inputs, densely packed anatomical structures, and low-contrast abnormalities.

### 3.3 Model training

Images with more than 30% occlusion due to eyelids, eyelashes, or artifacts were excluded from the dataset. The remaining images were normalized and randomly divided into training and testing sets in an 80:20 ratio. Three models—YOLOv8, YOLOv8+ convEMA, and YOLOv8+ convSimAM—were trained. Training was conducted for 200 epochs with a batch size of 16. The stochastic gradient descent (SGD) optimizer was employed with an initial learning rate of 0.01, a momentum of 0.937, and a weight decay coefficient of 0.0005.

## 4 Results

### 4.1 Evaluation metrics

To evaluate the performance of the proposed method, object detection was identified as the primary task, with a particular emphasis on the accurate localization and classification of various lesion types in DR images. Unlike conventional image classification, which merely determines the presence or absence of lesions, object detection simultaneously provides the spatial coordinates, class labels, and confidence scores for each detected lesion. Accordingly, we adopted precision, recall, and average precision (AP) as the principal evaluation metrics (Yang et al., 2023). For further performance evaluation, we computed the confusion matrix components, including true positive (TP): the number of lesion regions correctly predicted as positive; false positive (FP): the number of non-lesion regions incorrectly predicted as positive; and false negative (FN): the number of lesion regions mistakenly classified as negative.

Precision measures the proportion of correctly identified positive samples among all positive predictions, indicating the accuracy of lesion detection:

$$\text{Precision}=\text{TP }/\;\left(\text{TP}+\text{FP}\right).$$

Recall assesses the proportion of correctly identified positive samples among all actual positive cases, reflecting the model’s sensitivity to true lesions:

$$\text{Recall}=\text{TP }/\;\left(\text{TP}+\text{FN}\right).$$

AP represents the area under the precision–recall (PR) curve, capturing both detection accuracy and completeness. It is computed as follows:

$$AP=\sum_{i=1}^{n}\left(R_{i}‐R_{i‐1}\right)\cdot P_{i}.$$

Here, $P_{i}$ denotes the precision at the threshold i, and $R_{i}-R_{i-1}$ represents the incremental change in recall between two adjacent points on the PR curve.

### 4.2 Experimental results

**FIGURE 4:**
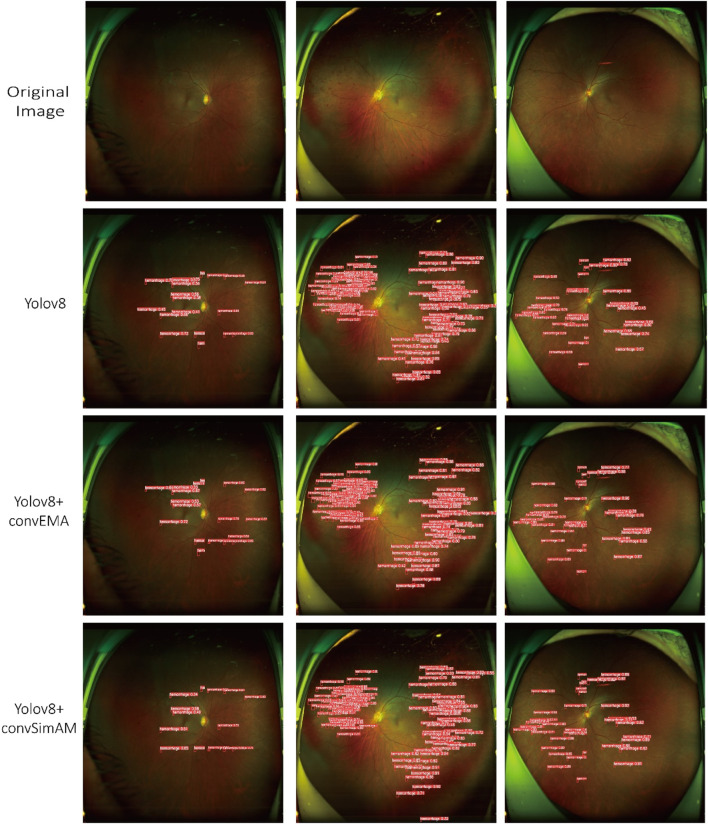
Three distinct fundus images are presented in three columns, with four rows corresponding to different detection models. Each red bounding box delineates a detected lesion along with its associated confidence score.

Based on model training and evaluation, we obtained detection results for four types of DR lesions: hemorrhages, hard exudates, cotton wool spots, and epiretinal membranes. These results were achieved using three model configurations: YOLOv8, YOLOv8+ convEMA, and YOLOv8+ convSimAM. The precision, recall, and AP metrics for each lesion type are presented in Table 1, providing a comprehensive comparison of model performance across the different lesion categories. Representative examples of lesion annotation outcomes are illustrated in Figure 4, demonstrating the model’s capability to localize and classify lesions in UWF fundus images.

**TABLE 1:** Comparison of precision, recall, and AP for lesion detection using different YOLOv8-based models.

Model	Detecting lesions	Precision	Recall	AP
YOLOv8	Hemorrhages	0.815	0.773	0.822
	Hard exudates	0.958	0.813	0.857
	Cotton wool spots	0.936	0.826	0.876
	Epiretinal membranes	0.847	0.900	0.899
YOLOv8+ convEMA	Hemorrhages	0.906	0.731	0.862
	Hard exudates	0.936	0.799	0.847
	Cotton wool spots	0.985	0.813	0.894
	Epiretinal membranes	0.797	0.924	0.978
YOLOv8+ convSimAM	Hemorrhages	0.910	0.739	0.868
	Hard exudates	0.955	0.804	0.853
	Cotton wool spots	0.990	0.832	0.899
	Epiretinal membranes	0.875	0.928	0.995

#### 4.2.1 Statistical analysis results

To further evaluate the statistical significance of performance differences among the models, paired t-tests were conducted. The comparison between YOLOv8 and YOLOv8+ convEMA revealed no significant difference (*p* = 0.362), indicating that the integration of convEMA alone may not substantially enhance lesion detection across all lesion types. In contrast, the comparison between YOLOv8+ convSimAM and YOLOv8+ convEMA demonstrated a statistically significant difference (*p* = 0.033), suggesting that convSimAM provides a superior enhancement for lesion detection. Furthermore, a significant difference was also observed between YOLOv8 and YOLOv8+ convSimAM (*p* = 0.039), supporting the effectiveness of spatial attention mechanisms in improving lesion localization and recognition.

#### 4.2.2 Lesion detection capability analysis

Based on the results presented in Table 1 and Figure 4, the YOLOv8 model demonstrated relatively high precision and recall in detecting hard exudates and cotton wool spots, with precision values of 0.958 and 0.936, respectively. However, its performance was slightly less effective in identifying hemorrhages and epiretinal membranes. The mean AP across different lesion categories ranged from approximately 0.85 to 0.89, indicating that the baseline YOLOv8 model possesses robust general detection capabilities. Nonetheless, the model exhibited limitations in capturing fine-grained lesion features, which led to occasional missed or incorrect detections, thereby highlighting potential areas for further optimization.

The YOLOv8+ convEMA model demonstrated significant improvements in the detection of cotton wool spots, achieving an AP value of 0.894. A notable enhancement was also observed in the detection of epiretinal membranes, with the AP increasing to 0.978. Although the precision for epiretinal membranes (0.797) was slightly lower than that of the baseline YOLOv8 model, the recall improved from 0.900 to 0.924, indicating an enhanced ability to capture more complex lesion patterns. These findings suggest that the integration of the convEMA attention mechanism has improved the model’s feature extraction capabilities, resulting in superior performance in detecting intricate lesion regions such as epiretinal membranes. However, the slight reduction in precision for certain lesion types indicates the presence of some false positives, highlighting the need for further refinement of the model.

The YOLOv8+ convSimAM model demonstrated superior performance in detecting cotton wool spots, achieving higher precision (0.990) and recall (0.832) than both the baseline YOLOv8 and the YOLOv8+ convEMA models. Notably, the AP for epiretinal membranes reached 0.995, indicating near-perfect performance. Furthermore, the model consistently achieved higher recall across all lesion categories compared to the other two models. The AP values were generally higher than those of the YOLOv8 and YOLOv8+ convEMA models, with the most significant gains observed in the detection of epiretinal membranes, highlighting the model’s enhanced capability to capture subtle and complex pathological features.

#### 4.2.3 Grad-CAM heatmap analysis

**FIGURE 5:**
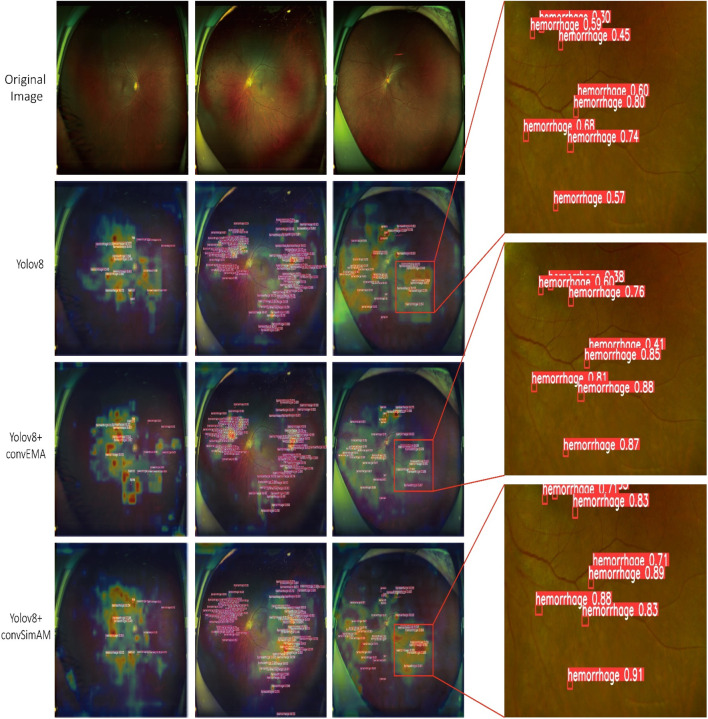
Visualization of model predictions and interpretability using Grad-CAM and object detection. The Grad-CAM heatmaps presented on the left highlight the image regions that significantly influence the model’s classification of the lesion, where red and yellow tones indicate higher relevance. On the right, each red bounding box delineates a detected lesion along with its associated confidence score.

As illustrated in Figure 5 and Table 1, the activation maps of the YOLOv8 model generally encompass the lesion areas; however, the activations appear relatively dispersed. Some regions do not accurately focus on the features of the lesions, and background interference remains prominent. For hard exudates, the activation areas mostly align with the target; however, in the case of epiretinal membranes, the boundaries of activation are less distinct, and certain regions may be overlooked. In contrast, the YOLOv8+ convEMA model exhibits more concentrated activation in the lesion regions, with significantly reduced activation intensity in non-lesion areas. The delineation of target features is moderately improved; however, the detection of hemorrhages remains somewhat suboptimal. Compared to YOLOv8, background noise is markedly diminished, and the overall activation distribution appears more uniform. The YOLOv8+ convSimAM model demonstrates the most distinct activation patterns, featuring clearly defined lesion boundaries and minimal background interference. This model exhibits the highest proficiency in extracting lesion-related features, particularly for epiretinal membranes, where contrast and edge details are significantly enhanced.

Overall, YOLOv8 serves as a stable baseline model, although its performance is somewhat limited. The integration of the EMA mechanism in YOLOv8+ convEMA significantly enhances the model’s capacity to identify target regions, leading to substantial improvements in the detection of complex lesions. Among the three models, YOLOv8+ convSimAM exhibits the best performance as its attention mechanism significantly enhances both detection precision and recall for specific lesion areas, making it the most comprehensive and effective model in this study.

## 5 Discussion

Our comparative analysis indicates that models enhanced with attention mechanisms generally outperform the baseline model in lesion detection. For hemorrhages, the baseline YOLOv8 model achieved relatively low precision (0.815) and had the lowest recall (0.773). In contrast, YOLOv8+ convEMA significantly improved precision to 0.906, although its recall decreased to 0.731. This decline may be attributed to the convEMA attention mechanism, which strengthens responses to prominent regions while suppressing peripheral areas, potentially leading to missed detections of small targets. Conversely, YOLOv8+ convSimAM improved both precision (0.910) and recall (0.739), achieving an AP of 0.868. This enhancement is likely due to SimAM’s spatial sensitivity mechanism, which bolsters responses to small regions and enhances recall performance.

In detecting hard exudates, all models demonstrated strong performance, with AP values exceeding 0.847. This suggests that these lesions are relatively easier to identify. The influence of attention mechanisms was minimal in this instance, and convEMA even exhibited a slight decline in performance, potentially due to an overemphasis on prominent features while neglecting boundary areas.

Regarding cotton wool spots, YOLOv8 achieved a recall of only 0.826, indicating that some detections were missed. In contrast, both YOLOv8+ convEMA and YOLOv8+ convSimAM significantly improved recall (up to 0.832) and precision (up to 0.990). Given the blurred boundaries and irregular distribution of cotton wool spots, attention mechanisms seem to enhance the model’s ability to recognize fine texture details—particularly convSimAM, which is more effective at responding to indistinct regions.

For epiretinal membranes, YOLOv8+ convEMA and YOLOv8+ convSimAM achieved recall rates of 0.924 and 0.928, respectively, which are significantly higher than the baseline YOLOv8 rate of 0.900. This indicates that the attention mechanisms substantially improve feature saliency in lesions characterized by a large size, complex structure, and poorly defined boundaries.

In summary, YOLOv8+ convSimAM is more effective for detecting small and dispersed targets, such as hemorrhages. For lesions with well-defined boundaries and strong contrast (e.g., hard exudates), the attention mechanisms offered limited additional benefits. However, for blurry or morphologically complex lesions—such as cotton wool spots and epiretinal membranes—the attention-enhanced models showed significant improvements in detection performance, with YOLOv8+ convSimAM demonstrating the most robust and consistent enhancements.

As illustrated in Figure 5, the heatmaps generated by Grad-CAM reveal distinct attention distribution patterns across models. The baseline YOLOv8 model exhibits dispersed activation regions, with considerable attention spread over irrelevant background areas. This leads to insufficient clarity in hemorrhage detection and suboptimal localization of lesions, even for hard exudates with relatively distinct visual characteristics. A possible explanation lies in the architecture of YOLOv8’s backbone components (e.g., C2f and SPPF), which, although designed for multi-scale feature extraction, lack an explicit mechanism to focus on lesion-specific regions. In DR images, lesions are typically small, low-contrast, and poorly defined, making their features easily diluted within the feature maps. Consequently, the model’s attention often diffuses to non-lesion areas, such as blood vessels and the optic disc.

In contrast, the heatmaps generated by the YOLOv8+ convEMA model demonstrate an enhanced focus on the core areas of lesions, particularly small-scale lesions such as hemorrhages. However, there is a noticeable lack of attention around the boundaries of these lesions, which impacts the accurate delineation of hard exudates that exhibit distinct morphological edges. This issue may arise from the characteristics of the convEMA module, which employs a convolutional channel attention mechanism in conjunction with exponential moving average smoothing. This enhances the weights of consistently high-activation regions within feature maps, allowing the model to “stably focus” on previously learned salient regions—especially small, bright lesions such as hemorrhages. However, since this mechanism is globally average-guided, it may suppress boundary or shape-related information, particularly in lesions with ambiguous or irregular contours.

The YOLOv8+ convSimAM model demonstrates the most comprehensive and well-concentrated activation maps. It successfully highlights both lesion centers and boundaries while effectively suppressing irrelevant background signals. These results confirm that SimAM, a parameter-free spatial attention mechanism, enhances the model’s capacity to analyze lesion features across multiple levels—including the size, structure, and edges.

The incorporation of attention mechanisms into the YOLOv8 architecture significantly enhanced lesion detection performance, suggesting that both the convEMA and convSimAM modules effectively guide the model to focus on lesion-specific regions rather than on background noise.

This study focuses on UWF fundus images, which capture a significantly broader retinal area (up to 200°) compared to conventional fundus photography. Aiello et al. (2024) reported a high concordance in DR outcomes between traditional 7-field imaging and UWF fundus images, demonstrating the clinical reliability of UWF fundus images. Additionally, Castellanos-Canales et al. (2024) found moderate agreement between clinical DR grading and UWF fundus images and between UWF fundus images and ultra-widefield fluorescein angiography. These findings suggest that UWF fundus images offer a more comprehensive view of the spatial distribution of DR lesions, making it superior to single-field conventional fundus photography for lesion characterization and diagnosis. Oh et al. (2021) developed a diagnostic model for UWF fundus images utilizing a ResNet-34 residual network, which extracted regions of interest (ROIs) centered on the optic disc and macula. Their model achieved an accuracy of 83.38% ± 0.47% in detecting. Wang et al. (2024) proposed a lesion detection model based on ultra-widefield fundus images using images and employing the R-CNN framework combined with ResNet-50 and a feature pyramid network (FPN). The model has a detection rate of 83.57%, 86.75%, and 87.23% for cotton wool spots, hard exudates, and hemorrhages, respectively.

In this study, we integrated attention mechanisms into the YOLOv8 architecture, which is lightweight and optimized for small object detection, striking a balance between inference speed and detection precision. Our model outperformed ResNet-based and traditional faster R-CNN architectures, particularly in identifying small lesions such as hemorrhages and cotton wool spots. Unlike existing systems such as IDx-DR (Khan et al., 2025) and DeepDR (Dai et al., 2021), which focus primarily on classification and severity grading, our approach emphasizes object-level detection of specific lesion types, including hemorrhages, cotton wool spots, and epiretinal membranes. The incorporation of convEMA and convSimAM attention modules significantly enhanced performance, especially for challenging targets such as hemorrhages and epiretinal membranes. Furthermore, the use of interpretable attention-based mechanisms enhances clinical confidence in AI-generated results—an advantage not provided by conventional end-to-end black-box models. It is important to note that the comparisons with other architectures, such as ResNet-based models and faster R-CNN, are based solely on results reported in the literature. Since no direct experimental benchmarking was conducted under unified datasets and conditions, this limitation of the present study will be addressed in future work.

Current research on YOLOv8 and its variants in ophthalmology has primarily focused on conventional color fundus photography. For instance, Amin et al. (2024) developed a novel model combining ResNet-18 and YOLOv8 to localize and grade lesions in NPDR images. Their model achieved average IoU scores of 0.95, 0.94, 0.96, and 0.95 for the optic disc, soft exudates, hard exudates, and microaneurysms, respectively. Moura et al. (2024) applied a YOLOv8-based deep learning framework for lesion segmentation and classification on the e-ophtha dataset, demonstrating high performance in detecting exudates and microaneurysms. To improve the detection of microaneurysms in fluorescein angiography, Zhang et al. (2023) employed SwinIR for super-resolution image reconstruction and inserted an additional detection layer between the neck and head of YOLOv8. Their enhanced MA-YOLO model achieved a recall of 88.23%, precision of 97.98%, F1 score of 92.85%, and AP of 94.62% for microaneurysm detection.

In contrast, our study demonstrates clear advantages in terms of image modality, detection framework, task comprehensiveness, and clinical deployability: (1) use of ultra-widefield fundus images, enabling a significantly broader view of the retina and improved visualization of lesion distribution; (2) joint detection of multiple lesion types, aligning with the progressive and multifactorial nature of DR pathogenesis; (3) lightweight model architecture, suitable for real-world clinical screening environments with limited computational resources; (4) incorporation of the parameter-free spatial attention module (SimAM), which effectively enhances boundary recognition and lesion saliency, particularly for diffuse or poorly defined features; and (5) integration of Grad-CAM for interpretability, which increases transparency in AI-driven diagnostics and fosters clinical trust. These features collectively underscore the strong clinical adaptability and practical value of our proposed method for AI-based ophthalmic screening platforms.

In this study, we present the first integration of two lightweight attention mechanisms—convEMA and convSimAM—into the backbone of the YOLOv8 architecture for the detection of DR lesions in UWF fundus images. This novel design balances detection precision, spatial perception, and model deployment efficiency, demonstrating strong task adaptability and structural innovation. Currently, there are no studies in the public literature that simultaneously introduce convEMA and SimAM modules into the YOLOv8 backbone network for medical image object detection, especially for UWF fundus images, which is even rarer. Prior research has primarily focused on color fundus photography (CFP) or fluorescein angiography (FFA), with most models designed for lesion classification or single-lesion detection tasks. These approaches often lack systematic modeling for multi-lesion localization in structurally complex and spatially extensive UWF fundus images. In our architecture, the convEMA module enhances the model’s stability in detecting small, low-contrast lesions, such as microaneurysms and small hemorrhages, while the SimAM module strengthens spatial attention, particularly improving sensitivity to blurred or peripheral lesions, such as peripheral hemorrhages and epiretinal membranes, which are common in UWF fundus images.

The combined use of these attention mechanisms with YOLOv8’s intrinsic multi-scale feature fusion significantly enhances contextual focus and spatial representation without sacrificing model efficiency. Experimental results demonstrate that our model outperforms the baseline YOLOv8 in detecting hemorrhages, cotton wool spots, and epiretinal membranes. Furthermore, Grad-CAM-based visualizations confirm that the attention mechanisms effectively redirect the model’s focus toward lesion-relevant regions, improving both lesion localization and boundary delineation. Compared to deeper architectures such as ResNet or SwinIR, our model offers superior deployability and practical applicability, making it well-suited for integration into real-world DR screening systems in clinical settings.

Despite the promising performance achieved in model structure optimization, integration of attention mechanisms, and application to UWF fundus images, this study has several limitations: (1) the absence of systematic horizontal comparisons with state-of-the-art (SOTA) models, such as DETR, YOLOv7-W6, and Swin Transformer, restricts a comprehensive evaluation of the model’s relative advantages; (2) the generalizability of the model remains inadequately validated as the dataset is sourced from a single center using uniform imaging devices and formats, which may introduce bias when applied across different equipment, populations, or imaging conditions; (3) the recall rate for certain small lesions is still suboptimal. Future work will focus on expanding data diversity, enhancing clinical collaboration, and further optimizing model precision, thereby establishing a solid foundation for the development of a generalized and deployable AI-based DR screening system.

## 6 Conclusion

This study addresses the challenge of multi-lesion detection of DR in UWF fundus images by proposing two enhancement strategies based on the YOLOv8 framework. Specifically, we integrated a channel-wise dynamic attention mechanism (convEMA) and a parameter-free spatial attention mechanism (convSimAM) into the backbone network. Through detection experiments targeting four representative DR lesions—hemorrhages, hard exudates, cotton wool spots, and epiretinal membranes—along with Grad-CAM-based visual interpretation, we demonstrated the effectiveness of attention mechanisms in enhancing lesion perception, refining spatial focus, and improving detection performance. Among the models evaluated, YOLOv8+ convSimAM achieved the best overall performance, exhibiting higher AP across all lesion types and producing clearer heatmaps concentrated on lesion regions. These characteristics make it particularly well-suited for detecting small, complex retinal lesions in fundus images. We hope this work contributes to the advancement of intelligent DR diagnosis research and provides clinicians with effective decision-support tools for the early diagnosis and prevention of vision loss in patients with DR.

## Data Availability

The original contributions presented in the study are included in the article/supplementary material; further inquiries can be directed to the corresponding author.
